# A high mitochondrial transport rate characterizes CNS neurons with high axonal regeneration capacity

**DOI:** 10.1371/journal.pone.0184672

**Published:** 2017-09-19

**Authors:** Romain Cartoni, Gulcin Pekkurnaz, Chen Wang, Thomas L. Schwarz, Zhigang He

**Affiliations:** 1 Department of Neurology, F.M. Kirby Neurobiology Center, Boston Children's Hospital, Boston, Massachusetts, United States of America; 2 Department of Neurology, Harvard Medical School, Boston, Massachusetts, United States of America; 3 Department of Neurobiology, Harvard Medical School, Boston, Massachusetts, United States of America; Imperial College London, UNITED KINGDOM

## Abstract

Improving axonal transport in the injured and diseased central nervous system has been proposed as a promising strategy to improve neuronal repair. However, the contribution of each cargo to the repair mechanism is unknown. DRG neurons globally increase axonal transport during regeneration. Because the transport of specific cargos after axonal insult has not been examined systematically in a model of enhanced regenerative capacity, it is unknown whether the transport of all cargos would be modulated equally in injured central nervous system neurons. Here, using a microfluidic culture system we compared neurons co-deleted for PTEN and SOCS3, an established model of high axonal regeneration capacity, to control neurons. We measured the axonal transport of three cargos (mitochondria, synaptic vesicles and late endosomes) in regenerating axons and found that the transport of mitochondria, but not the other cargos, was increased in PTEN/SOCS3 co-deleted axons relative to controls. The results reported here suggest a pivotal role for this organelle during axonal regeneration.

## Introduction

Neurons from the CNS normally fail to regenerate their axons after an injury. Recently, it has been demonstrated that specific genetic manipulations could achieve robust CNS axons regeneration *in vivo*. For example, the deletion of the Phosphatase and tensin homolog (PTEN) and the Suppressor of cytokine signaling 3 (SOCS3) or the over-expression of c-Myc have revealed a previously unknown plasticity of the CNS after axonal injury [1–3]. These mutants with high regenerative capacity constitute an opportunity to study the key biological events occurring during axonal regeneration. However, though these models have been extensively characterized *in vivo*, the cell biology of these mutants has been sparsely studied. Using the conditioning lesion paradigm, Mar and colleagues showed that the central branches of DRG axons globally increased their vesicular transport during regeneration [4]. It is unknown however whether CNS neurons regulate their axonal transport similarly during axonal regeneration or if some vesicles and organelles are preferentially targeted. Crucial events for axonal regeneration such as microtubule stabilization and the formation of an active growth cone [5–7] require high energy consumption [8,9], which can be provided by mitochondria. Hence it is likely that changes to mitochondrial transport will be of special importance in injured axons to achieve re-growth by providing mitochondria to crucial areas. Mitochondria are dynamic organelles whose traffic is especially important in neurons whose axons extend over considerable distances [10–12]. Axonal transport in CNS neurons has been mostly studied in axons of cultured neurons in conditions where the axons have not been specifically injured after the initial establishment of the culture. The lack of good high-regeneration models has limited knowledge of the regulation of transport during axonal regeneration. Although *in vivo* imaging has made possible the study of vesicular transport in living animals [4,13], comparing the transport of several cargos in equivalent conditions remains a challenging task *in vivo*. Here, using microfluidic chambers, we systematically studied the transport of three cargos, mitochondria, synaptic vesicles and late endosomes, in regenerating axons of cultured CNS neurons co-deleted for PTEN and SOCS3 [14]. Each of these organelles undergoes microtubule-based axonal transport mediated by kinesin and dynein motors [12,15,16]. We found that unlike the transport of synaptic vesicles and late endosomes, the transport of mitochondria correlated with the high regeneration capacity of PTEN^-/-^; SOCS3 ^-/-^ neurons.

## Materials and methods

### Transgenic mice

All experiments procedures were performed in compliance with animal protocols approved by the IACUC at Boston Children’s Hospital. The PTEN^f/f^; SOCS3^f/f^; Synapsin Cre line was obtained by breeding PTEN^f/f^; SOCS3^f/f^ [14] mice with mice from a Synapsin Cre line acquired from Jackson Laboratories. The STOP^f/f^; TdTomato; Synapsin Cre line was obtained from Jackson Laboratories.

### Cortical neurons culture and transfection

18 days after the mating of PTEN^f/f^; SOCS3 ^f/f^; Synapsin Cre mice and PTEN^f/f^; SOCS3 ^f/f^ mice, pregnant females were euthanized by decapitation. A piece of tail of each embryo was kept for genotyping and cortex from each embryo was treated individually. Cortical neurons were isolated following standard procedures. Briefly, cortices were chemically dissociated in papain solution (Worthington Biochemicals) for 10 min at 37°C followed by two washes in trypsin inhibition solution (Sigma T9253) and mechanical dissociation using P1000 plastic tips in Neurobasal medium (Life Technology). Dissection, dissociation and washes were done in HBSS (Gibco 1470–112) with 45% glucose and kynurenic acid (Sigma K3375) (HBSS, kyuneric acid 10mM, 100mM Hepes, 100mM MgCl2). Cells were plated in microfluidic chambers (RD450, Xona Microfluidic) placed on rectangular glass cover slips (Corning, 24x40) coated with poly-l-lysine hydrobromide (Sigma). Neurons were cultured in Neurobasal medium (Life Technology) supplemented with B-27 (Life Technology), L-Glutamine (Life Technology) and Penicillin/Streptomycin (Life Technology). Half of the medium was replaced at day in vitro 3 (DIV3). Plasmid DNA transfections were performed using Lipofectamin 2000 (Invitrogen) following the manufacturer’s procedure. We used 0.3μg of DNA per microfluidic chamber. Neurons were transfected at DIV 5. At DIV6 axons were severed as described in [17] at the point where they exited from the microfluidic grooves into the axonal chamber. 20h post injury the culture medium was replaced by Hibernate E low fluorescence (BrainBites) to maintain cell viability in CO2- free conditions during live imaging.

### Immunohistochemistry

For immunohistochemistry of cortical neurons, DIV 6–7 neurons were fixed with 4% PFA and immunostained using standard procedures. Briefly, neurons were fixed directly in microfluidic chambers with 4% PFA and incubated 1 hour in blocking solution (BSA 3%, Triton 0.5% in PBS) followed by an overnight incubation with primary anti-Tuj1 antibody at 1/200 (Covance), anti-MAP2 (1/200, Sigma) and anti-GFAP (1/200, Dako). After a PBS wash, secondary antibodies conjugated to Alexa-488 or Alexa-594 (1/400- Life Technology)were added for 1 hour. Cover slips were mounted using Fuoromount-G (SouthernBiotech).

### Axonal regeneration assay quantification

Before injury, each device was imaged in bright field and the number of axons emerging in the axonal compartment was counted every 100μm up to 600μm from the exit of the microgrooves. Immediately post injury, devices were imaged to ensure no intact axons remained. 20h post injury we imaged the devices and counted the number of regenerating axons every 100μm up to 600μm from the exit of the microgrooves. The average percentage of regenerating axons was calculated using the number of intact axons for each distance as the baseline (100%) for a particular device. The corresponding number of regenerating axons of the same device 20h post injury was then used to determine the percentage of regenerating axons.

### Live imaging

Time-lapse movies were acquired on a PerkinElmer Spinning Disc confocal microscope equipped with a temperature-controlled chamber at 37°C and with a 20 x / N.A. 0.90 oil objective. Images were captured every 2 s over 60 frames as previously described [18] with laser power set to 30% for each channel to minimize damage. For both cortical neurons and retina explant experiments, a 80–120 μm portion of axon located 150μm from the axon’s tip was selected for analysis. Dendrites could not extend through the grooves of the microfluidic chambers and therefore allowed us selectively to examine axons[19]. For the experiments where neurons were double-transfected with MitodsRed2 and EYFP-Synaptophysin, the red and green channels were recorded simultaneously. For axons transfected with Rab7-GFP and DsRed2 as a axonal marker, only the green channel was recorded. Volocity software (PerkinElmer) was used for live recording. We used a custom-made Image J macro for kymograph-based motility analysis as described in [18]. ImageJ raw data were extracted as excel spreadsheets (Microsoft Excel).

### Statistical analysis

Statistical analysis was performed using GraphPad Prism version 6.0a for Mac OS X (GraphPad Software, Inc., La Jolla, CA, USA). For vesicular transport analysis, the normality of the distribution of each dataset was first tested using the D’Agostino & Pearson omnibus test, the Shapira-Wilk test and the KS test. Because the moving frequency and total distance travelled of the studied vesicles failed to pass these tests, the non-parametric Mann-Whitney *U* test was chosen.

## Results

### An *in vitro* platform to study axonal transport in injured high regeneration capacity CNS neurons

**Fig 1:**
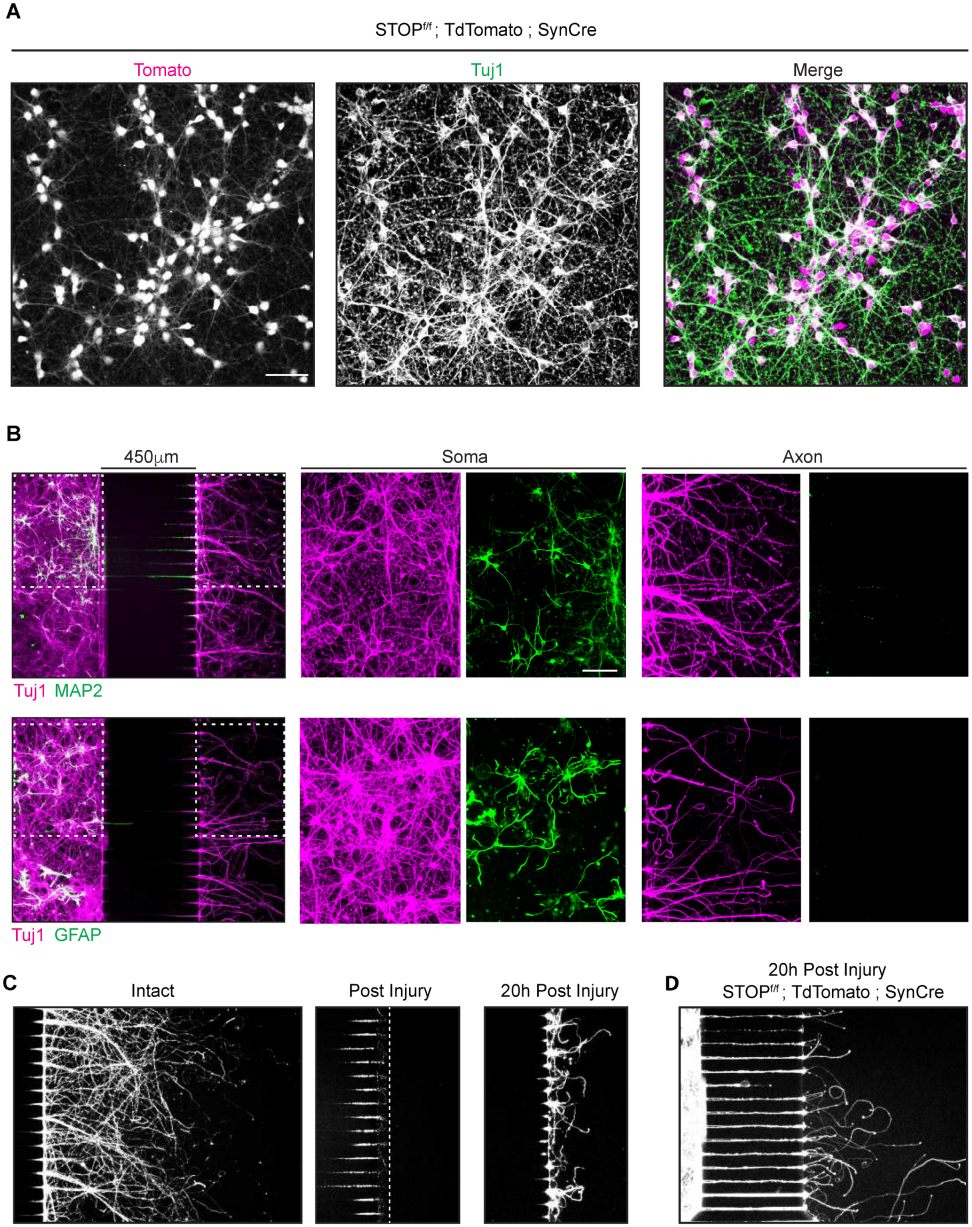
Characterization of Synapsin Cre in neurons cultured in microfluidic chambers. (A) Immunohistochemistry of cortical neurons (DIV6) isolated from Synapsin Cre; stop^f/f^ TdTomato transgenic mice. Anti Tuj1 antibody was used as a neuronal marker. TdTomato (magenta in the merged image) is present in almost all the neuronal cell bodies. Scale bar = 50μm. (B) Immunohistochemistry using Tuj1 (axonal marker, magenta), MAP2 (dendrite marker, green first row) or GFAP (glial cell marker, green second row) antibodies on E18 mouse cortical neurons culture (DIV7) in microfluidic chambers. Higher magnifications images of somal and axonal compartments are shown in the second and third columns. Neurons were plated in the chamber on the left and their axons grew through the grooves in the center section to emerge in the axonal chamber at the right. 450 μm microgrooves allows a complete isolation of axons from dendrites and glia as indicated by the absence of those markers from the right-hand chamber. Antibodies typically did not reach inside the microgrooves unless explicitly caused to enter (not shown), which is why grooves remain largely dark. Scale bar = 100μm (C) Tuj1 immunohistochemistry of E18 mouse cortical neurons cultures (DIV7) in microfluidic chambers: No Injury (left), immediately after injury (center) and 20 h after injury (right). (D) E18 mouse cortical neurons cultures from Synapsin Cre; stop^f/f^ TdTomato embryo culture fixed 20 h post axonal injury.

The double deletion of the genes encoding PTEN and SOCS3 has been shown to induce a very high regeneration phenotype after nerve injury *in vivo* [14]. We reasoned that this well-established model of high regenerative capacity would constitute a suitable tool to study the specificity of axonal transport during axonal regrowth. To study *in vitro* the effect of the double deletion of PTEN and SOCS3 on axonal transport, we generated a transgenic mouse line double-deleted for PTEN and SOCS3 in CNS neurons by breeding PTEN^f/f^; SOCS3^f/f^ mice with transgenic mice expressing the Cre recombinase under the pan-neuronal promoter Synapsin (PTEN^f/f^; SOCS3^f/f^; SynCre). We confirmed that Synapsin-Cre successfully recombined floxed alleles in our culture conditions by isolating E18 cortical neurons from the STOP^f/f^; TdTomato; SynCre mouse (Fig 1A).

**Fig 2:**
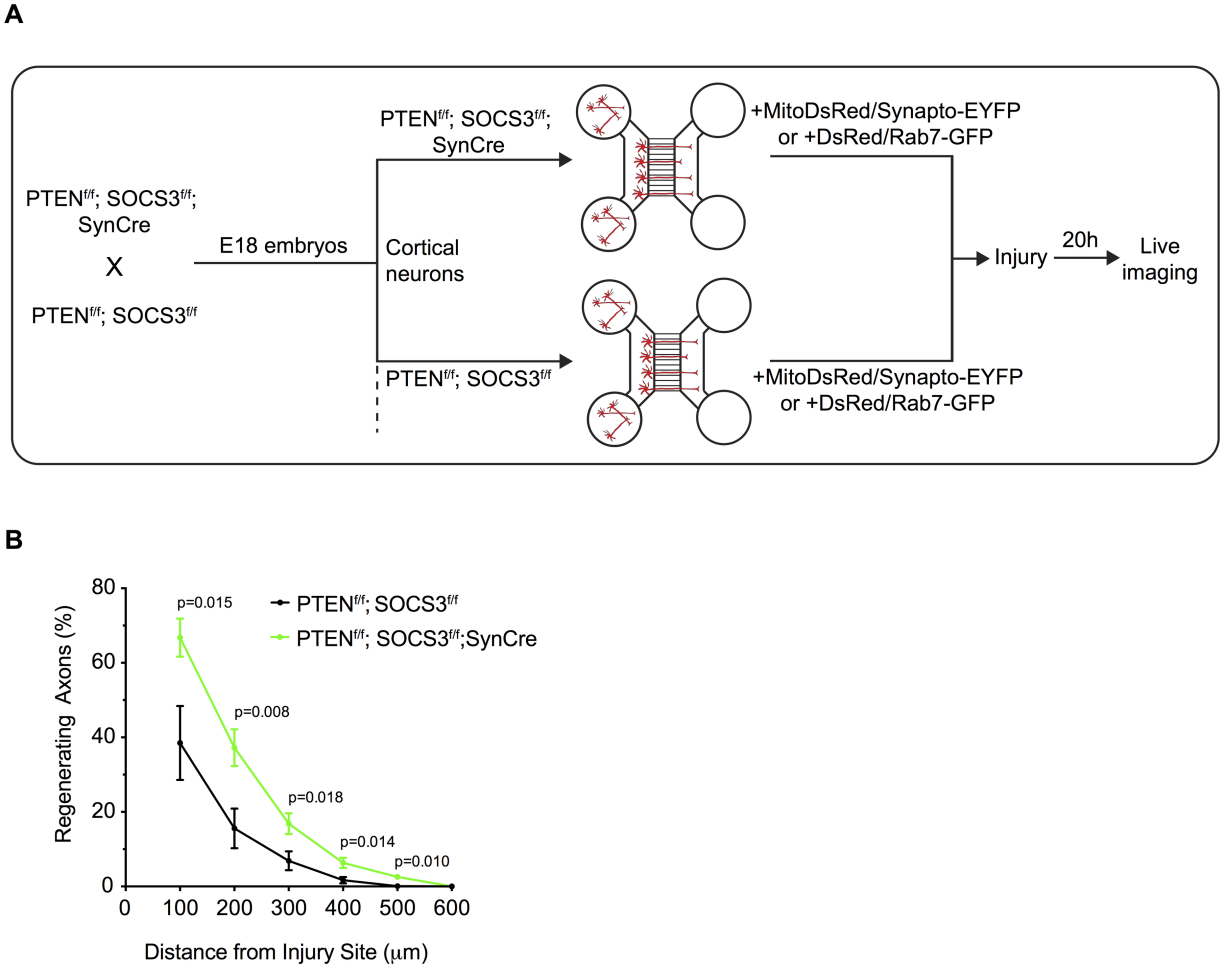
Deletion of PTEN and SOCS3 improves axonal regeneration of E18 cortical neurons. (A) Schematic of the *in vitro* platform to study the axonal transport in regenerating PTEN^-/-^; SOCS3^-/-^ cortical neurons. PTEN^-/-^; SOCS3^-/-^ E18 cortical neurons were obtained by breeding PTEN^f/f^; SOCS3^f/f^ mice with the PTEN^f/f^; SOCS3^f/f^; Synapsin Cre (SynCre) mice. The cortex of each embryo was processed individually so that each microfluidic chamber was seeded with neurons from a single embryo. Thereby PTEN^f/f^; SOCS3^f/f^; SynCre neurons were compared to PTEN^f/f^; SOCS3^f/f^ from littermate embryos. All neurons were cotransfected with MitoDsred2 and EYFP-Synaptophysin or Rab7-GFP. (B) Quantification of *in vitro* axonal regeneration of PTEN^-/-^; SOCS3^-/-^ and control cortical neurons 20h post injury. n = 9–11 microfluidic cultures of individual embryos from 5 independent experiments. Two tailed Student’s Unpaired *t*-test.

In order to measure mitochondrial transport during axonal regeneration we took advantage of a microfluidic chambers culture system. These devices not only allow the separation of axons from dendrites and glial cells but also permit one to induce an axonal injury without affecting the cell body ([19] and Fig 1B and 1C). We further showed by inducing an axonal injury to the STOP^f/f^; TdTomato; SynCre neurons that the expression of the Cre recombinase persisted during axonal regeneration 20 hours post injury (Fig 1D). To validate our system, we then tested whether the double deletion of PTEN and SOCS3 could further improve the intrinsic axonal regeneration capacity of embryonic cortical neurons. To this end, we compared the axonal re-growth capacity of cortical neurons double-deleted for PTEN and SOCS3 obtained from SynCre; PTEN^f/f^; SOCS3^f/f^ embryos to control neurons from the Cre-negative PTEN^f/f^; SOCS3^f/f^ littermate embryos (Fig 2A).

Reminiscent of the results that showed high regeneration of PTEN^-/-^; SOCS3^-/-^ neurons *in vivo* [14], the double deletion of PTEN and SOCS3 increased axonal regeneration of E18 cortical neurons 20 hours post injury (Fig 2B). By coupling high regeneration capacity in cultured neurons with the microfluidic chamber system we established a reliable in vitro platform to study axonal transport in injured CNS neurons and evaluate the correlation of regeneration capacity with organelle dynamics.

### PTEN^-/-^; SOCS3^-/-^ regenerating axons are characterized by a high mitochondrial transport rate

**Fig 3:**
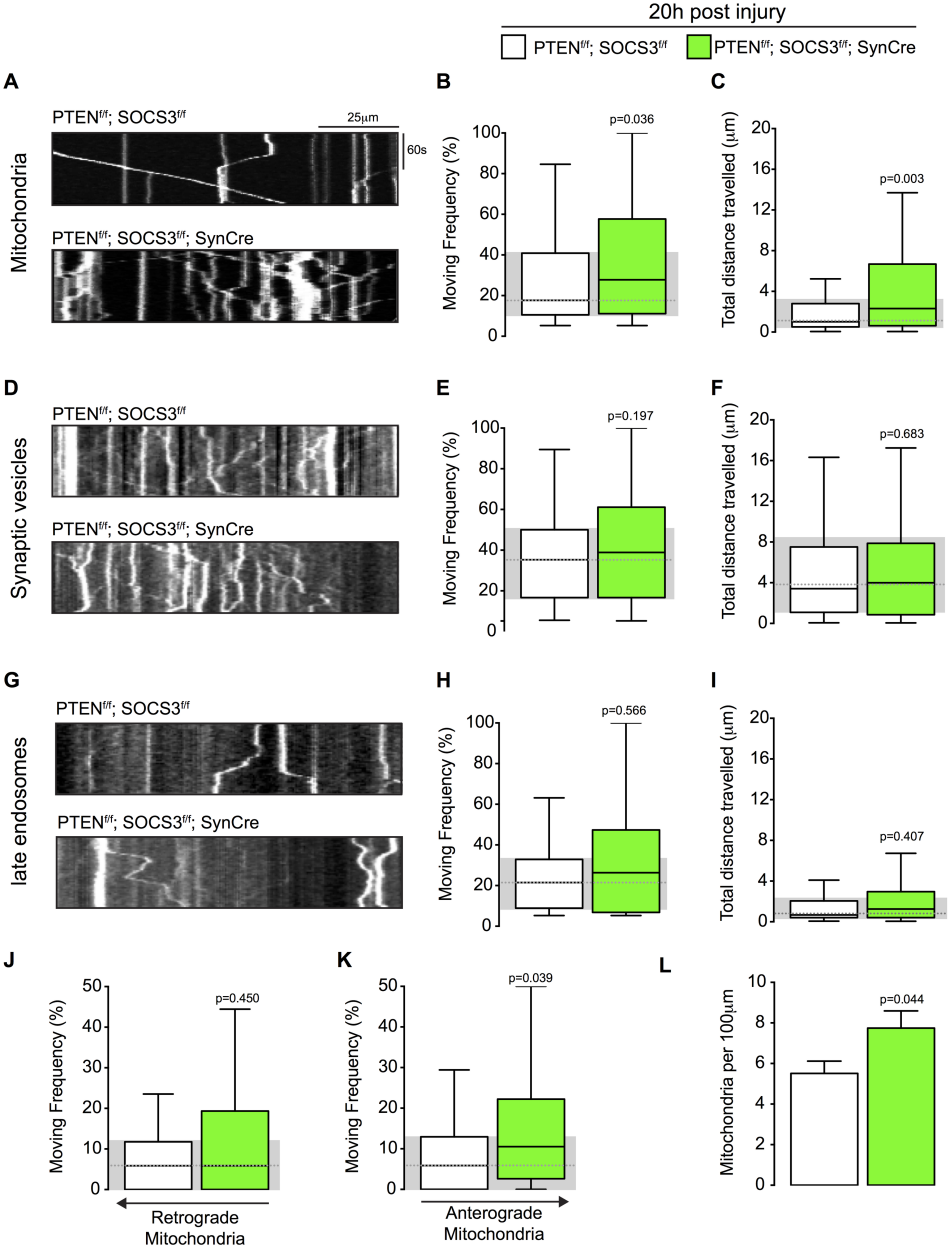
Mitochondrial transport is increased in regenerating axons co-deleted for PTEN and SOCS3. (A) Representative kymographs from live imaging of mitochondria in regenerating axons from PTEN^f/f^; SOCS3^f/f^ and PTEN^f/f^; SOCS3^f/f^; SynCre neurons 20 h post injury. Consecutive line scans of the axon were arrayed top to bottom so that the y-axis of the kymograph represents the time and the x axis the position of the object studied. Stationary objects therefore appear as vertical lines and motile ones as diagonals. (B, C) Box plot showing the moving frequency of motile mitochondria (B) and their distance travelled (C) in regenerating axons of the indicated genotypes. Co-localization between MitoDsRed and Mitotracker confirmed that the vast majority of the mitochondria in the transfected cells were labeled (S1 Fig). Mann-Whitney *U* test on the number of mitochondria. n = 136–223 mitochondria from 18–22 axons and 4–6 individually cultured embryos from 2 independent litters. (D-F) Representative kymographs (D) and quantification of the moving frequency (E) and distance travelled (F) from live imaging of EYFP-synaptophysin-positive synaptic vesicle precursors in regenerating axons of the indicated genotypes. Mann-Whitney *U* test. n = 153–226 synaptic vesicles, 12–17 axons, 4–5 individually cultured embryos from 2 independents experiments. (G-I) Representative kymographs (G) and box plots of moving frequency (H) and total distance travelled (I) from live imaging of Rab7-GFP positive endosomes in regenerating axons of indicated genotype. (Mann-Whitney *U* test. n = 69–105 late endosomes, 13–14 axons, 5–4 individually cultured embryos from 2 independents experiments. (J and K) Retrograde *(J)* (toward cell body) and anterograde *(K)* (toward axon’s tip) moving frequencies of the mitochondria analyzed in (A). Mann-Whitney *U* test. (L) Mitochondrial densities in the axons analyzed in (A). Two tailed Student’s Unpaired *t*-test. Data in all the box plots are represented with a box that delimitates the lower (Q1) and the upper quartile (Q3) of the distribution. Horizontal line indicates the median (Q2) and whiskers indicate the maximum and minimum of the distribution.

We then asked whether the improved axonal regeneration observed in PTEN^-/-^; SOCS3^-/-^ neurons was coupled with increased axonal transport and if the transport of vesicular cargos was similarly affected. We co-transfected PTEN^-/-^; SOCS3^-/-^ and control cortical neurons, with the mitochondrial marker MitoDsRed2 and Synaptophysin-EYFP, a marker for a pool of synaptic vesicle precursors. We separately co-transfected neurons of these genotypes with Rab7-GFP in order to follow endosomal transport and DsRed2 as an axonal marker (Fig 2A). As with mitochondria (S1 Movie), we observed that Synaptophysin-EYFP positive cargo present different sizes, probably due to the transport of vesicle aggregates (S2 Movie). Similarly, Rab7-GFP vesicles display a range of sizes (S3 Movie). In all conditions, all cargo were included in the quantification regardless of their size. All cultures were subjected to axonal injury at the point where axons emerged from the microfluidic grooves by aspirating the medium from the axonal compartment [19]. Using live imaging microscopy of the regenerating axons 20 hours post injury, we analyzed two transport parameters for each organelle class. We measured the percentage of time the organelles spent in motion (moving frequency) and the total distance they travelled during the recording. Axons contain a stationary pool of mitochondria and also a motile pool; mitochondria in the motile pool alternate between movements and pauses [11]. If the double deletion of PTEN and SOCS3 did not affect the proportion of these pools (S2A Fig), we found by examining the fraction of time this motile pool was in motion that the motile mitochondria had a significantly higher moving frequency in regenerating PTEN^-/-^; SOCS3^-/-^ axons compared to control axons (Fig 3A and 3B, S1 Movie).

The deletion of PTEN and SOCS3 had also a significant effect on the total distance travelled by the motile mitochondria in the regenerating axons (Fig 3C). We also observed a modest effect on the speed of retrograde transport (S2D Fig). Whereas the PTEN^-/-^; SOCS3^-/-^ deletion had a profound impact on mitochondrial transport, it did not influence the transport of synaptophysin-positive synaptic vesicles (Fig 3D–3F, S2 Movie) or Rab7-GFP-labelled organelles (Fig 3G–3I, S3 Movie). Interestingly, the higher moving frequency of motile mitochondria was largely due to an increase in the anterograde (*i*.*e*. toward growth cone) direction (Fig 3J and 3K). Additionally, we observed that PTEN^-/-^; SOCS3^-/-^ regenerating axons have a higher mitochondrial density than controls (Fig 3L). We noted also that Rab7-GFP positives vesicles were more abundant in regenerating PTEN^-/-^; SOCS3^-/-^ axons suggesting an enhancement of endocytosis (data not shown).

### Basal mitochondrial transport is increased in PTEN^-/-^; SOCS3^-/-^ neurons

**Fig 4:**
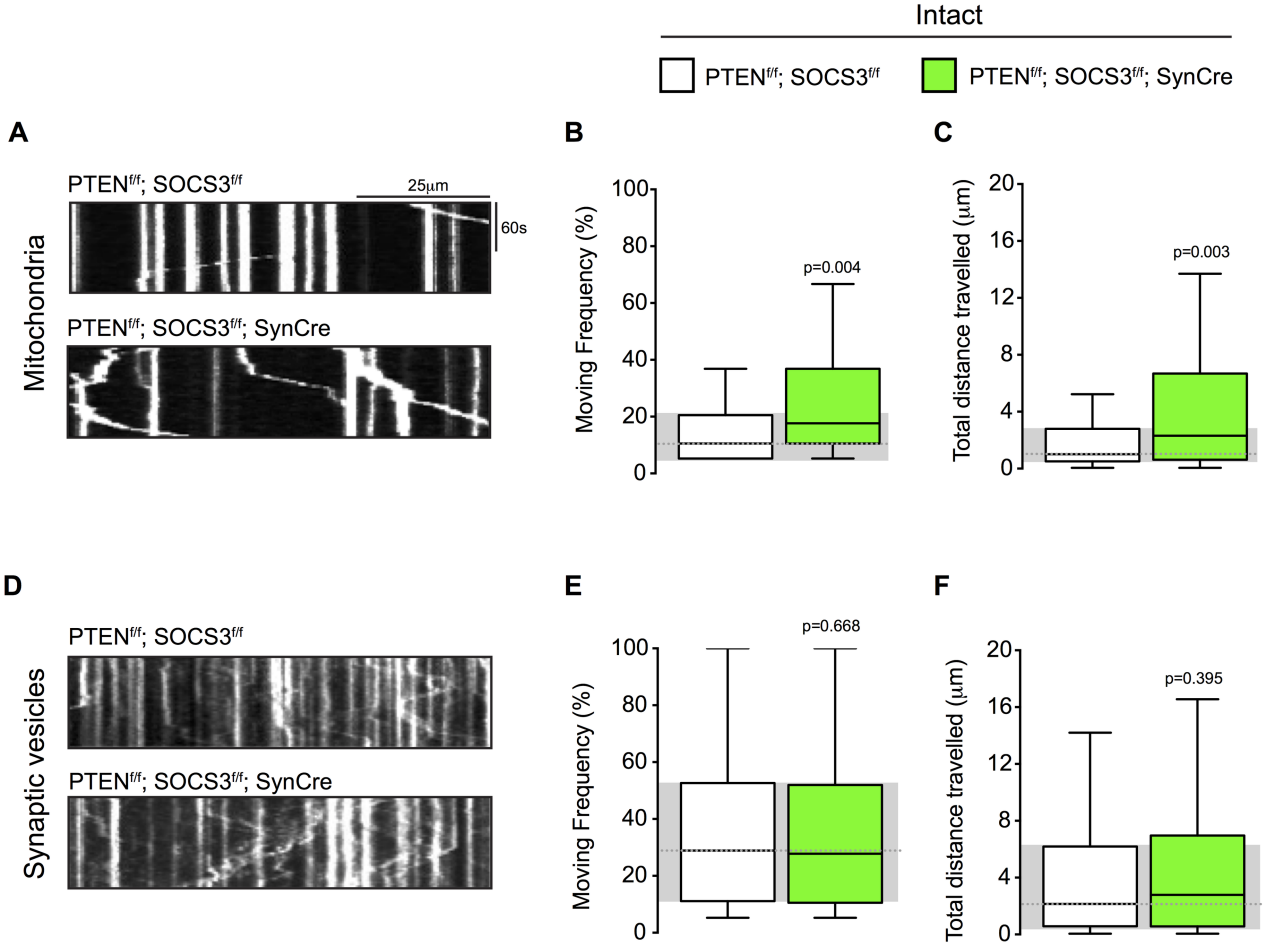
PTEN and SOCS3 co-deletion increased mitochondrial transport in cultured neurons whose axons were not severed. (A-C) Representative kymographs (A) and quantification of moving frequency (B) and distance travelled (C) from live imaging of mitochondria in PTEN^f/f^; SOCS3^f/f^ and PTEN^f/f^; SOCS3^f/f^; SynCre in intact axons. Mann-Whitney *U* test on the number of mitochondria. n = 117–118 mitochondria from 13–14 axons and 4–5 individually cultured embryos from 2 independent experiments. (D-F) Representative kymographs (D) and quantification of moving frequency (E) and distance travelled (F) from live imaging of synaptophysin-positive synaptic vesicle in intact axons of indicated genotype. Mann-Whitney *U* test. n = 123–212 synaptophysin-positive synaptic vesicles from 13–14 axons and 4–5 individually cultured embryos from 2 independent experiments.

To determine if the high mitochondrial transport rate observed in regenerating PTEN^-/-^; SOCS3^-/-^ axons was injury dependent, we studied mitochondrial transport in intact PTEN^-/-^; SOCS3^-/-^ axons. We again co-transfected MitoDsRed and Synaptophysin-EYFP, but did not injure the axons (Fig 2A). The moving frequency of the motile mitochondrial in intact PTEN^-/-^; SOCS3^-/-^ axons, was significantly higher than in control axons (Fig 4A and 4B) and mitochondria in PTEN^-/-^; SOCS3^-/-^ axons also travelled further (Fig 4C).

Consistent with our results in injured axons (Fig 3A–3I), we did not observed an increase of the synaptophysin-positive synaptic vesicles transport in intact PTEN^-/-^; SOCS3^-/-^ axons (Fig 4C–4E). These results suggest that a high mitochondrial transport rate might prime the neuron for a robust response to axonal injury. Similarly to the injured conditions, we did not observe a change in the proportion of the two pools (motile and stationary) (S2B Fig) and the direction of the mitochondrial transport was skewed towards anterograde transport (S2C Fig).

## Discussion

By testing the axonal transport rate of three cargos (mitochondria, synaptic vesicles and late endosomes) in a well-established model of CNS neurons with high regenerative capacity, we showed that axonal transport is not globally increased, but rather that mitochondrial transport is specifically modulated and is likely to contribute to the robust axonal regeneration of the PTEN^-/-^;SOC3^-/-^ neurons. Furthermore, we demonstrated that microfluidic chambers represent a valid alternative to animal use. We cannot rule out that other untested cargos might be similarly affected by the co-deletion of PTEN and SOCS3 during axonal regeneration. However, this result provides evidence that the axonal transport of cargos is not uniformly upregulated in conditions that support the re-growth program of a neuron. Our results contrast with those reported in a study of the DRG neurons [4]. Lysosomes, synaptophysin-positive synaptic vesicles, APP-positive vesicles, and mitochondria all showed a higher transport rate in DRG neurons in response to pre-conditioning, than in control neuron [4]. It is possible that both the type of neuron and the type of injury will affect the response in axonal transport. Furthermore, it would be of great interest to observe *in vivo* two or more cargos simultaneously in the same axons of the same mice in order to fairly compare transport parameters. Consistent with our findings that suggest a specific role for mitochondrial transport during axonal regeneration, Zhou and colleague recently knocked down the mitochondrial anchoring protein Syntaphilin and found that this selective increase of mitochondrial transport was sufficient to facilitate axonal regeneration [20]. Furthermore, in c-elegans, axonal regeneration depends on the localization of mitochondria to injured axons [21]. Our study also shows a higher mitochondrial density in regenerating PTEN^-/-^; SOCS3^-/-^ axons (Fig 3L). This higher density is a logical consequence of increased transport into the axon; we do not presently know whether the improved regeneration results from the increased density or from increased motility per se. In addition to increasing their mitochondrial transport rate during axonal regeneration, we found that uninjured PTEN^-/-^; SOCS3^-/-^ neurons have a higher basal mitochondrial transport rate in culture than wild type neurons. Thus a high mitochondrial transport rate may constitute a prerequisite for efficient axonal repair rather than a stress response mechanism activated after injury. We note however that axons are already in a regenerating state by virtue of the neurons having been placed in culture. Therefore, we cannot rule out that the mitochondrial transport rate reported here in intact wild type neurons (*i*.*e*. without medium aspiration of the axonal side causing specific axonal injury) is itself a result of an injury-dependent mechanism. Because PTEN and SOCS3 regulate many targets, finding the effector protein(s) by which their loss promotes mitochondrial transport remains an open question. Results from our lab using optic nerve crush suggest, however, that PTEN^-/-^; SOCS3^-/-^ neurons transcriptionally up regulate the mitochondrial protein Armcx1 and that this protein is sufficient to increase mitochondrial transport and promote axonal re-growth of retinal ganglion cells *in vivo* [22]. Furthermore, Armcx1 over expression also increases mitochondrial transport in axons of E18 cortical neurons. It is therefore possible that the high mitochondrial transport rate described here in PTEN^-/-^; SOCS3^-/-^ neurons is Armcx1 dependent. We note however that that PTEN^-/-^; SOCS3^-/-^ cortical neurons increase their mitochondrial transport rate by enhancing the moving frequency of the motile mitochondria (Fig 3A–3C) rather then mobilizing stationary mitochondria (S2A and S2B Fig) as observed when Armcx1 is over expressed in retinal ganglion cells[22]. Therefore, we need to consider the possible cell type specificity of targets affected by PTEN and SOCS3 deletion. Additional work is needed to decipher the regulation of mitochondria not only in different types of neurons but also at different developmental stages. Together, these studies and others highlight the numerous ways a neuron can modulate the transport of their mitochondria and the significance of that modulation for axon growth and regeneration [21,23]. Because numerous neurodegenerative diseases have been linked to mitochondrial transport defects [24–26], the results reported here, provide an important basis for future work on possibilities of enhancing mitochondrial functions during neuronal repair and axonal regeneration.

## Supporting information

S1 Fig Co-localization between MitoDsRed transfected neurons and Mitotracker dye. (TIF)

S2 Fig Mitochondrial transport parameters. (TIF)

S1 Movie PTEN and SOCS3 co-deletion increased mitochondrial transport 20h post injury. (MOV)

S2 Movie PTEN and SOCS3 co-deletion does not increase the transport of synaptophysin-positive synaptic vesicles 20h post injury. (MOV)

S3 Movie PTEN and SOCS3 co-deletion does not increase the transport of late endosomes 20h post injury. (MOV)
