# Supplementary figures and images for: A high mitochondrial transport rate characterizes CNS neurons with high axonal regeneration capacity
Source: PLoS One. 2017 Sep 19;12(9):e0184672. doi: 10.1371/journal.pone.0184672 (PMC5604968; doi:10.1371/journal.pone.0184672)

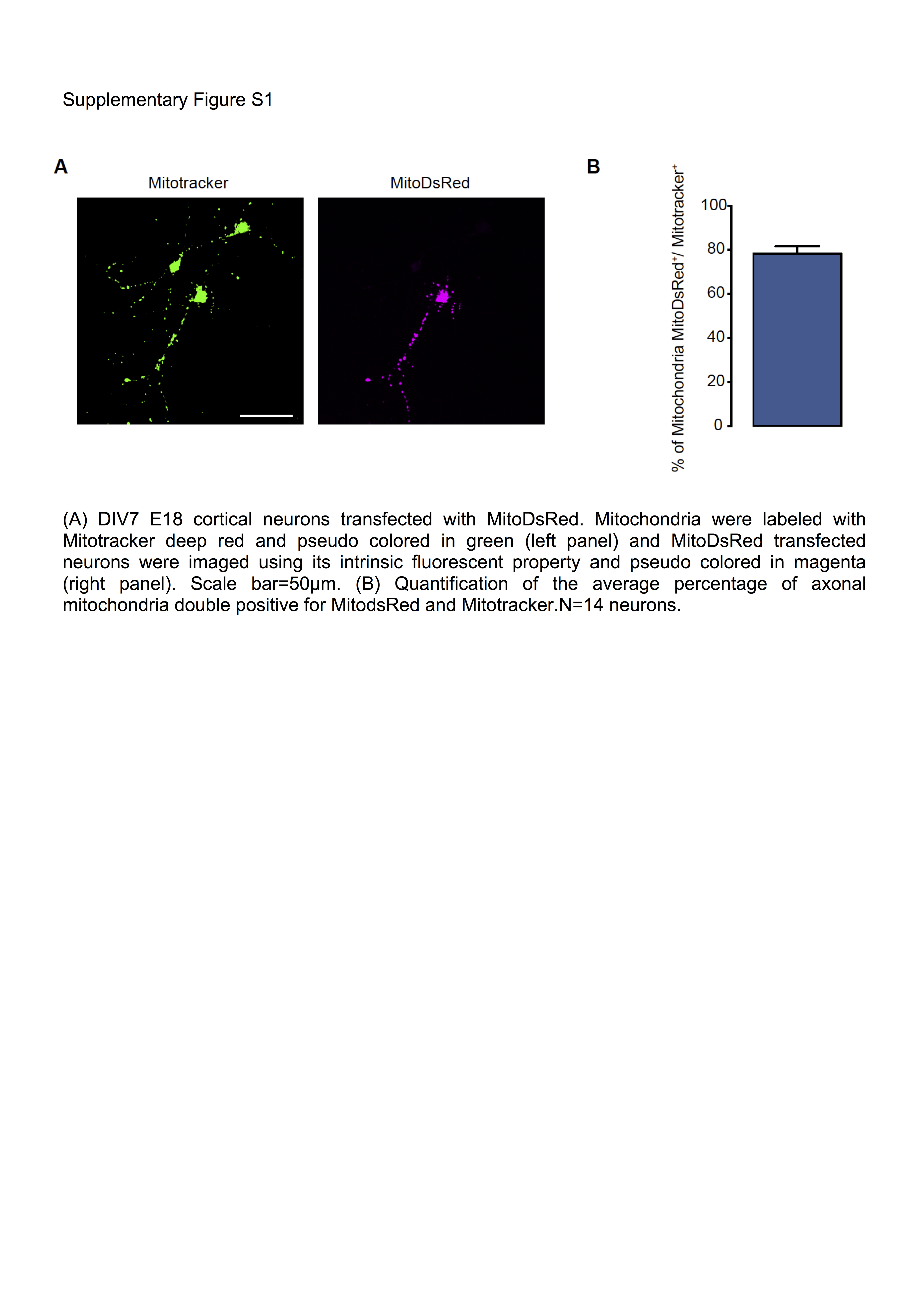

Supplement: S1 Fig — (TIF) [file pone.0184672.s001.tif]

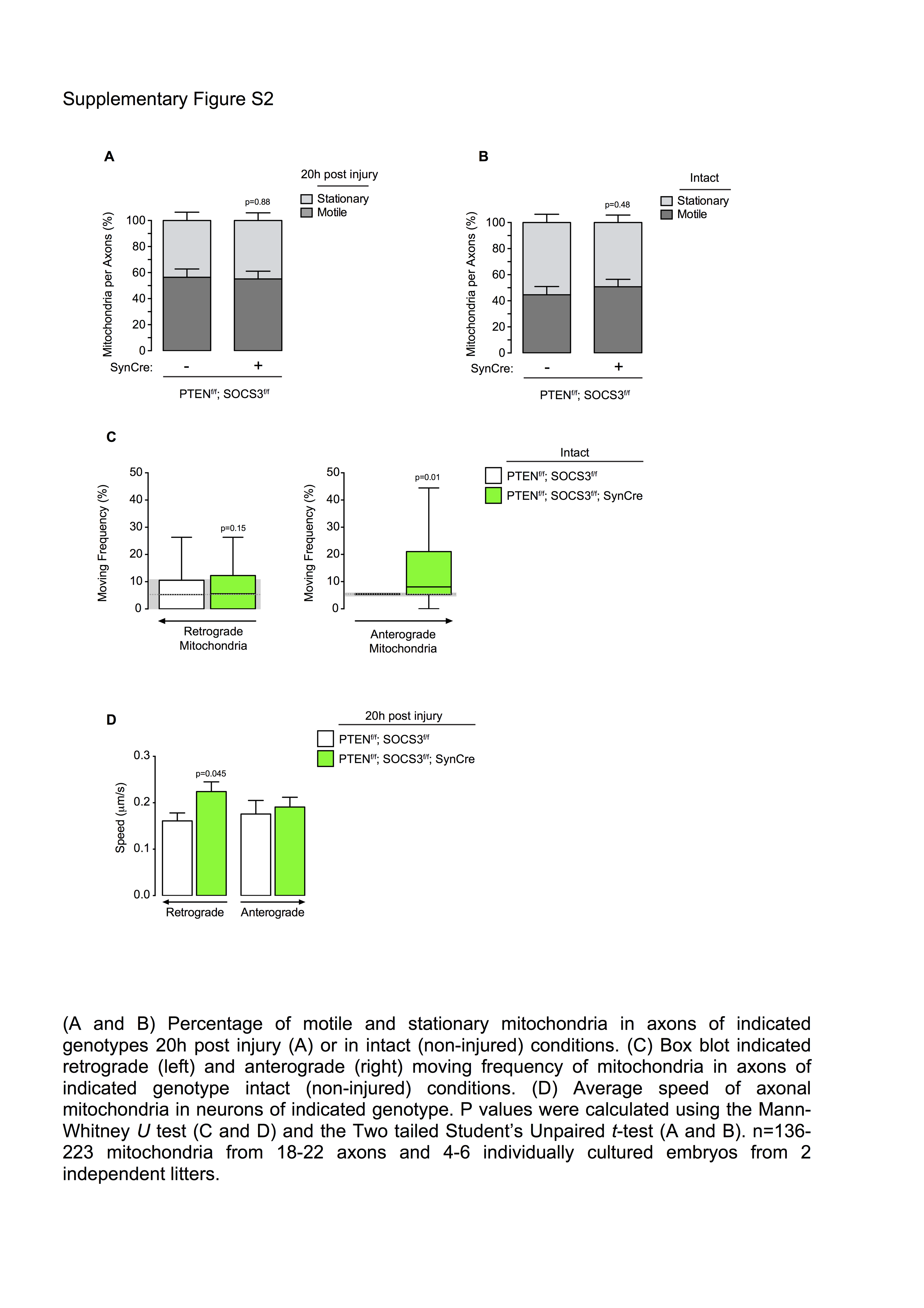

Supplement: S2 Fig — (TIF) [file pone.0184672.s002.tif]
